# Kinematic and Functional Gait Changes After the Utilization of a Foot Drop Stimulator in Pediatrics

**DOI:** 10.3389/fnins.2019.00732

**Published:** 2019-07-30

**Authors:** Kiran K. Karunakaran, Rakesh Pilkar, Naphtaly Ehrenberg, Katherine S. Bentley, JenFu Cheng, Karen J. Nolan

**Affiliations:** ^1^Center for Mobility and Rehabilitation Engineering Research, Kessler Foundation, West Orange, NJ, United States; ^2^Department of Biomedical Engineering, New Jersey Institute for Technology, Newark, NJ, United States; ^3^Department of Physical Medicine and Rehabilitation, Rutgers – New Jersey Medical School, Newark, NJ, United States; ^4^Children’s Specialized Hospital, Mountainside, NJ, United States

**Keywords:** functional electrical stimulation, foot drop, hemiplegia, stroke, cerebral palsy, gait, pediatric rehabilitation

## Abstract

Foot drop is one of the most common secondary conditions associated with hemiplegia post stroke and cerebral palsy (CP) in children, and is characterized by the inability to lift the foot (dorsiflexion) about the ankle. This investigation focuses on children and adolescents diagnosed with brain injury and aims to evaluate the orthotic and therapeutic effects due to continuous use of a foot drop stimulator (FDS). Seven children (10 ± 3.89 years) with foot drop and hemiplegia secondary to brain injury (stroke or CP) were evaluated at baseline and after 3 months of FDS usage during community ambulation. Primary outcome measures included using mechanistic (joint kinematics, toe displacement, temporal-spatial asymmetry), and functional gait parameters (speed, step length, time) to evaluate the orthotic and therapeutic effects. There was a significant correlation between spatial asymmetry and speed without FDS at 3 months (*r* = 0.76, *p* < 0.05, df = 5) and no correlation between temporal asymmetry and speed for all conditions. The results show orthotic effects including significant increase in toe displacement (*p* < 0.025 *N* = 7) during the swing phase of gait while using the FDS. A positive correlation exists between toe displacement and speed (with FDS at 3 months: *r* = 0.62, *p* > 0.05, without FDS at 3 months: *r* = 0.44, *p* > 0.05). The results indicate an orthotic effect of increased dorsiflexion and toe displacement during swing with the use of the FDS in children with hemiplegia. Further, the study suggests that there could be a potential long-term effect of increased dorsiflexion during swing with continuous use of FDS.

## Introduction

Children with hemiplegia due to stroke or cerebral palsy (CP) have unilateral motor deficits due to paralysis or weakness. Currently, 500,000 children under the age of 18 with CP (Prevalence of Cerebral Palsy, 2018) and 1 in 1,600 neonate, and 13 per 100,000 older children are affected by stroke each year in the United States (Bernson-Leung and Rivkin, 2009). Foot drop is one of the common secondary conditions associated with hemiplegia and is characterized by the inability to lift the foot (dorsiflexion) about the ankle due to paralysis or weakness of the peroneal and anterior tibialis muscles (Stewart, 2008). Foot drop, can affect the ability of the toes to clear the floor during the swing, and can also impair stability during the stance. Reduced toe clearance impedes the ability to walk efficiently and may cause reduced functional mobility. One of the most common deficits for individuals with hemiplegia is decreased dorsiflexion during swing leading to deficits in walking (Winters et al., 1987). Consequently, compensatory mechanisms like steppage gait (Dubin, 2014), hip hiking (Kerrigan et al., 2000; Dubin, 2014), toe walking(Dubin, 2014), etc. are used to successfully ambulate. These pathological deviations from the healthy walking result in slower walking speed (Sheffler and Chae, 2015), shorter step length (Sheffler and Chae, 2015), increased risk of falls due to kinematic changes (Winters et al., 1987) and decreased inter-limb temporal and spatial symmetry (Patterson et al., 2010; Nadeau, 2014).

Currently, custom molded ankle foot orthosis (AFO), a passive compensation device, is predominantly used to clinically treat foot drop. AFOs provide support and stability to the foot and ankle during stance phase, and provide clearance to the foot during the swing phase as the foot is always maintained at a predetermined angle (Lehmann et al., 1983; Gök et al., 2003). Though AFOs provide the necessary orthotic effect for foot drop, they can restrict the passive and active ankle range of motion (ROM) (Gök et al., 2003). This restricted ROM may lead to reduced muscle activity (Geboers et al., 2002).

Recent research is focusing on restoring function through the use of functional electrical stimulation (FES) (Cauraugh et al., 2010; Khamis et al., 2018). An FES device or foot drop stimulator (FDS) electrically stimulates the peroneal nerve to activate the peroneal and tibialis anterior muscles to dorsiflex the foot during swing phase (Selzer et al., 2006). Research suggests that FES can not only provide sufficient dorsiflexion to clear the foot during the swing but may also provide the user with rehabilitative benefits, as it would not restrict the user’s passive and active ROM (Comeaux et al., 1997; Van Der Linden et al., 2008; Laufer et al., 2009). Previous research on adults with foot drop has shown significant orthotic and therapeutic improvements in walking speed (Burridge et al., 1997; Voigt and Sinkjaer, 2000), temporal and spatial gait symmetry (Comeaux et al., 1997; Lin et al., 2006; Balasubramanian et al., 2007; Patterson et al., 2008), spasticity, and energy consumption with the use of FES (Yan et al., 2006). In addition, using neurophysiological measures, Stein et al. have shown that the therapeutic effects in functional outcome measures are in response to the neuroplastic changes resulting from the use of FES (Everaert et al., 2010). Extensive evidence supporting the efficacy of FES in treating foot drop is available for adults but similar evidence is not currently available in children. Previous research on children with foot drop have shown changes in functional outcome measures such as increased walking speed (Cauraugh et al., 2010; Prosser et al., 2012; Prenton et al., 2018), increased stride length (Pool et al., 2014), reduced use of compensatory mechanisms (Pool et al., 2014), and decreased mean energy expenditures (El-Shamy and Abdelaal, 2016) with the use of FES, but they have not investigated the kinematic and inter-limb asymmetry changes, and its association with functional outcomes due to immediate (orthotic) and continuous (therapeutic) use of FES. Though walking speed reflects overall gait performance, it does not depict underlying impairments in joint mechanisms and inter-limb coordination during gait, and their recovery. Winter et al. observed that ankle joint angle changes in young adults, howsoever small, could significantly improve toe clearance (Winter, 1992). Research in percutaneous stimulation and FES in children and adolescents have shown increased dorsiflexion with no significant changes in speed, but have shown changes in musculature(Orlin et al., 2005). Therefore, understanding the joint kinematic changes could provide insight into the mechanistic effects of FDS even when evidence is lacking in functional outcomes. Studying mechanistic changes could provide quantitative information about sub-clinical kinematic outcomes (Hausdorff et al., 2001).

Temporal and spatial symmetry have previously been studied to understand pathological walking patterns (Patterson et al., 2010). Studies have reported that temporal asymmetry is a significant predictor of hemiparetic walking performance such as walking speed and falls in adults (Roth et al., 1997; Patterson et al., 2010). In healthy individuals, the variability in temporal and spatial parameters is minimal (Patterson et al., 2010). In contrast, individuals with hemiplegia present with higher asymmetry between their limbs, leading to the ambulatory deficits (Patterson et al., 2010). Understanding the asymmetry characteristics during gait will help improve the design of rehabilitation interventions for individuals with deficits in functional ambulation (Wall and Turnbull, 1986). Quantifying the change in asymmetry would help us further understand the effect of using FDS on functional/clinical outcomes such as falls, etc. (Hausdorff et al., 2001).

The objective of this exploratory investigation is to evaluate the orthotic and therapeutic effects of using FDS for children and adolescents by evaluating mechanistic and functional outcomes. Orthotic effect is defined as the improvement while using the device (FDS is active on the affected limb) and the therapeutic effect is defined as any lasting effect once the device is removed (FDS is inactive or removed). The secondary objective will explore the adaptive effect, which is defined as the changes in orthotic effect over time. The primary hypotheses were that the use of a FDS will result in increased toe clearance (displacement) and temporal-spatial symmetry. The secondary hypotheses were that toe displacement and temporal-spatial symmetry will correlate with gait speed. This investigation will provide preliminary evidence for utilization of FDS in pediatric rehabilitation.

## Methodology

### Participants

Eleven participants with foot drop and hemiplegia secondary to brain injury (age: between 5 and 17 years) were recruited. Only data from seven participants (four male and three female) were available for analysis because kinematic data were not available, participants did not complete both time points or participants were discharged due to FDS non-compliance. The demographics of the seven participants are shown in Table 1. Additional inclusion criteria stated that participants: (1) were > 6 months post diagnosis of brain injury; (2) had no history of injury or pathology in the unaffected leg within the past 90 days; (3) were able to walk independently for 10 m without any assistive device; (4) had no history of Botulinum toxin injection to the lower limbs within 3 months of enrollment and no Botulinum toxin injection during the course of study; (5) had no severe cognitive or communication impairment; and (6) were not involved in any other interventions or were not using any devices/medications that might interfere with the study. Individuals with additional orthopedic, neuromuscular, or neurological pathologies that would interfere with their ability to walk were not included in this study. The protocol was approved by the Kessler Foundation Institutional Review Board (IRB), and consent was obtained before study participation from each participant’s parent/guardian (in addition assent was obtained for participants > 13 years). All participants used an AFO for community ambulation prior to their participation in the study, and they were encouraged to use the FDS without an AFO during the course of the study.

**TABLE 1:** Subject demographics.

**Subject**	**Condition**	**Affected side**	**Gender**	**Age at consent (years)**	**Height at baseline (m)**	**Weight at baseline (Kg)**	**Height at 3 months (m)**	**Weight at 3 Months (Kg)**	**Years since injury**
1	CP	Right	F	16	1.45	45.00	1.47	47.70	16
2	CP	Right	F	9	1.51	40.28	1.55	41.4	9
3	Stroke	Right	F	6	1.22	26.10	1.24	26.55	5
4	CP	Left	M	7	1.30	30.60	1.33	34.20	7
5	Stroke	Left	M	15	1.83	71.21	1.83	70.65	15
6	Stroke	Right	M	8	1.35	23.85	1.35	23.85	8
7	CP	Right	M	10	1.57	52.65	1.60	56.70	10
Mean ± (SD)				10.14 ± 3.89	1.46 ± 0.20	41.38 ± 16.77	1.48 ± 0.199	43.01 ± 16.79	

### Foot Drop Stimulator

**FIGURE 1:**
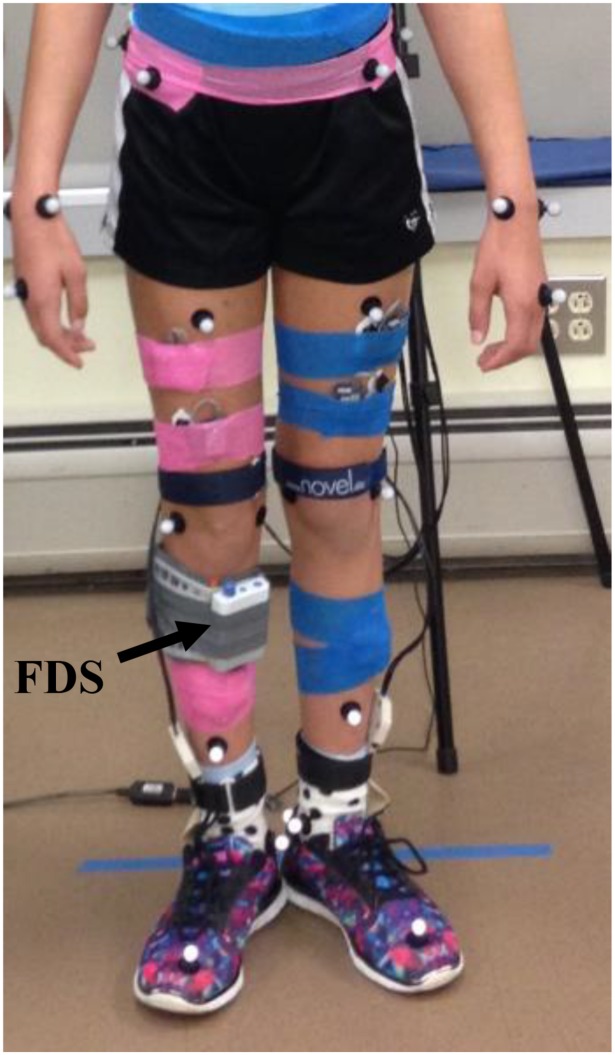
Subject wearing the FDS.

Functional electrical stimulation was provided through a commercially available FDS device (WalkAide^®^, Innovative Neurotronics, Austin, TX, United States). The WalkAide device uses surface electrodes in a single cuff located at the proximal end of the tibia about the peroneal nerve to stimulate during the swing phase of the gait cycle with customized frequency (17–33 Hz), pulse width (25–300 μs), and intensity of the generated electrical pulse to produce the desired dorsiflexion during gait. The FDS timing is controlled by a tilt sensor and accelerometer that will determine when to stimulate the peroneal nerve by monitoring the wearer’s leg position during gait. The small device [87.9 g, 8.2 cm (H) × 6.1 cm (W) × 2.1 cm (T)] is attached to a molded cuff located just below the knee, secured with a latch, and properly aligned using anatomical landmarks and visual indicators (Figure 1).

### Procedures

All participants completed up to eight study visits including screening, fitting, and device adjustment visits. These were followed by three data collection sessions (baseline, 1 month and 3 months). At their first visit, participants were screened and consented by a member of the study staff. Each participant was then fitted with a FDS by a licensed clinician at their second visit. During the device adjustment visits, a clinician provided training including review of device usage (donning and doffing), electrode placement, stimulation intensity adjustments, and a review of safety precautions. Following the adjustment visits, baseline data collection was conducted, and at the completion of the baseline session, all participants were given the device to use for community ambulation. All participants were instructed to use the device for everyday community ambulation for the entire duration of the study. Follow-up data collection was conducted at 1-month, and 3-month time points to assess the effect of continuous use of the device during community ambulation. This study was part of a larger investigation, and data from the baseline and 3-month visits were used for data analysis as 1-month data was not available for some participants. During each data collection session, retroreflective markers were placed on anatomical landmarks of the participants based on a modified Helen-Hayes marker configuration (Davis et al., 1991). Gait kinematics in the sagittal, frontal, and transverse planes were collected using a 12-camera motion capture system (Motion Analysis Corporation, Santa Rosa, CA, United States) at 60 Hz. At every data collection session, all seven participants performed in two conditions: (1) with FDS; and (2) without FDS, for a total of up to 24 walks (12 walking trials per condition). The order was randomized between with and without FDS condition for all subjects. During each trial, all participants were instructed to walk approximately 10 m on an unobstructed walkway at a self-selected speed. All participants wore shoes and did not wear any other assistive device during the data collection sessions. Participants were monitored during each trial and permitted to rest or take breaks at any time during testing to minimize the effects of fatigue.

### Data Processing and Analysis

All marker positions and joint angle data were collected, processed, and exported using Motion Analysis’s Cortex software (Motion Analysis Corporation, Santa Rosa, CA, United States) and analyzed using MATLAB (The Mathworks Inc., Natick, MA, United States). The Cartesian and joint kinematics data was exported from Cortex and filtered using a fourth order low pass filter. MATLAB was used to analyze the gait trajectories for each session with and without the FDS. The exported data was further divided into gait cycles, with a gait cycle being defined as the period from ground contact of one foot to the subsequent ground contact of the same foot. A minimum of 10 and a maximum of 25 gait cycles per condition, and per session were available for each subject and used for data analysis. The outcome measures and the analyses are shown in Table 2.

**TABLE 2:** Outcome measures.

**Outcome measure**	**Description**	**Statistical analysis**
Sagittal plane kinematics	The knee and ankle angles were computed for each gait cycle and averaged for each subject	
Toe displacement	The toe displacement was calculated as the maximum of the normalized difference in Cartesian y position of the toe (*ytoe*) and the heel (*yheel*) as shown in the equation below (y position data was normalization with respect to the foot position on the floor during stance). The average toe displacement was calculated for each subject M⁢a⁢x⁢(n⁢o⁢r⁢m⁢a⁢l⁢i⁢z⁢e⁢d⁢(y⁢t⁢o⁢e-y⁢h⁢e⁢e⁢l)) (1)	Shapiro-Wilk test (*p* > 0.05) of normality showed that data was normal. Repeated measures analysis of variance (ANOVA) was used to determine the effect of FDS and the effect of duration of use (Baseline to 3 months). Further, a *post hoc* test (pairwise comparison) with Bonferroni correction was performed to determine the difference between the conditions
Walking speed	The average walking speed was computed as the linear distance with respect to time to complete a gait cycle	Shapiro–Wilk test (*p* > 0.05) of normality showed that data was normal. Repeated measures analysis of variance (ANOVA) was used to determine the effect of FDS and the effect of duration of use (Baseline to 3 months) on walking speed
Step length	The average step length for each gait cycle was computed as the forward linear displacement between foot contact of the ipsilateral leg to foot contact of the contralateral leg during each gait cycle	Shapiro–Wilk test (*p* > 0.05) of normality showed that data was normal. Repeated measures analysis of variance (ANOVA) was used to determine the effect of FDS and the effect of duration of use (Baseline to 3 months) on step length
Temporal measures	Total time was computed as the time between foot contact of one leg to the subsequent foot contact of the same leg. The average total time was computed for each gait cycle. Further, average swing time for each subject during each condition was computed as the time between the foot off the floor of one leg to foot contact of the same leg during the gait cycle	Shapiro–Wilk test (*p* > 0.05) of normality showed that data was normal. Repeated measures analysis of variance (ANOVA) was used to determine the effect of FDS on total time. Further, a *post hoc* test (pairwise comparison) with Bonferroni correction was performed to determine the difference between the conditions A Friedman non-parametric test was used to determine the effect of FDS and the effect of duration of use (Baseline to 3 months) on swing time, as the data did not pass the test for normality (*p* < 0.05) using Shapiro–Wilk test
Temporal-spatial symmetry	The swing and stance time of each foot during each gait cycle was computed, and the following ratios were used to compute the temporal asymmetry: Temporal⁢swing⁢stance⁢symmetry=(swing⁢time)/(stance⁢time) (2) Equation 2 was computed for both affected and unaffected limbs $\mathrm{Overall}\,\mathrm{temporal}\,\mathrm{asymmetry}=\mathrm{abs}\,\left(1-\left(\frac{\mathrm{affected}\,\mathrm{swing}\,\mathrm{stance}\,\mathrm{symmetry}}{\mathrm{unaffected}\,\mathrm{swing}\,\mathrm{stance}\,\mathrm{symmetry}}\right)\right)\;(3)$ The step length of each foot was computed for each gait cycle, and the spatial asymmetry ratio was calculated as follows: $\mathrm{Spatial}\,\mathrm{asymmetry}=\mathrm{abs}\,\left(1-\left(\frac{\mathrm{unaffected}\,\mathrm{step}\,\mathrm{length}}{\mathrm{affected}\,\mathrm{step}\,\mathrm{length}}\right)\right)\;(4)$	Shapiro–Wilk test (*p* > 0.05) of normality showed that data was normal. Repeated measures analysis of variance (ANOVA) was used to determine the effect of FDS and the effect of duration of use (Baseline to 3 months) on temporal asymmetry. Further, a *post hoc* test (pairwise comparison) with Bonferroni correction was performed to determine the difference between the conditions A Friedman non-parametric test was used to determine the effect of FDS and the effect of duration of use (Baseline to 3 months) on spatial asymmetry, as the data did not pass the test for normality (*p* < 0.05) using Shapiro–Wilk test of normality

## Results

### Sagittal Kinematics

**FIGURE 2:**
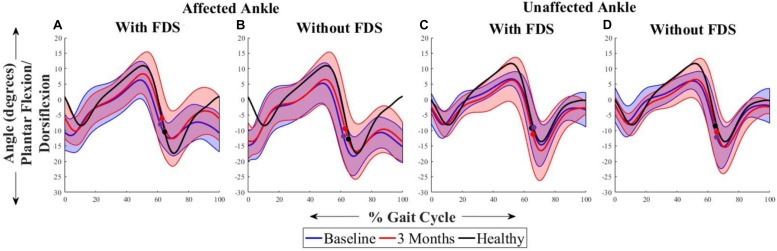
Mean ± Standard deviation (SD) ankle plantarflexion/dorsiflexion of the **(A)** affected ankle with FDS, **(B)** affected ankle without FDS **(C)** unaffected ankle with FDS and **(D)** unaffected ankle without FDS, at baseline and 3 months of all participants. The black line represents the mean ankle plantarflexion/dorsiflexion of one representative healthy child. The dots represent the start of swing phase.

Figure 2 shows the average sagittal plane kinematics of the ankle joint for the unaffected and affected legs with and without FDS for both sessions. At 3 months compared to baseline with FDS (adaptation effect), there is an increased mean dorsiflexion during swing on the affected side as well as increased plantar flexion during the beginning of stance phase (Figure 2A) but at 3 months compared to baseline without FDS (therapeutic effect), there is only a small increase in mean dorsiflexion during swing (Figure 2B) on the affected side. Also, at 3 months, increased mean dorsiflexion was observed during swing with FDS compared to without FDS (Figures 2A,B), and at baseline a small increase in mean dorsiflexion with FDS compared to without FDS (orthotic effect). No difference was observed between baseline and 3 months on the unaffected side with and without FDS (Figures 2C,D). However, the mean dorsiflexion angle during swing of the affected ankle was close to the unaffected ankle with use of FDS at 3 months (Figures 2A,C), and the ankle angle profile with FDS at 3 months is similar to that of the healthy control (Figure 2A).

**FIGURE 3:**
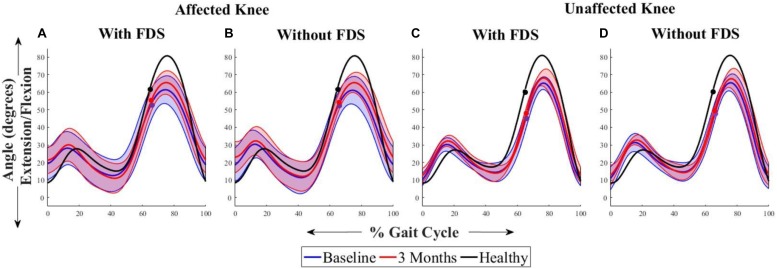
Mean ± Standard deviation (SD) knee plantarflexion/dorsiflexion of the **(A)** affected knee with FDS, **(B)** affected knee without FDS **(C)** unaffected knee with FDS and **(D)** unaffected knee without FDS, at baseline and 3 months of all participants. The black line represents the mean knee plantarflexion/dorsiflexion of one representative healthy child. The dots represent the start of swing phase.

Figure 3 shows the average sagittal plane kinematics of the knee for the unaffected and affected legs with FDS for both sessions. The results show that at 3 months compared to baseline with FDS, there is a small increase in mean flexion at the beginning of swing on the affected side (Figure 3A) but was within standard deviation (SD). At 3 months compared to baseline without FDS, there is a small increase in mean flexion at the beginning of swing (Figure 3B) on the affected side but was within SD. No difference was observed at baseline or at 3 months in knee flexion/extension between with and without FDS on the affected (Figures 3A,B) and unaffected sides (Figures 3C,D). Increased flexion was observed in the knee angles of both legs in the healthy control participant compared to the mean knee angles of participants with disability.

**FIGURE 4:**
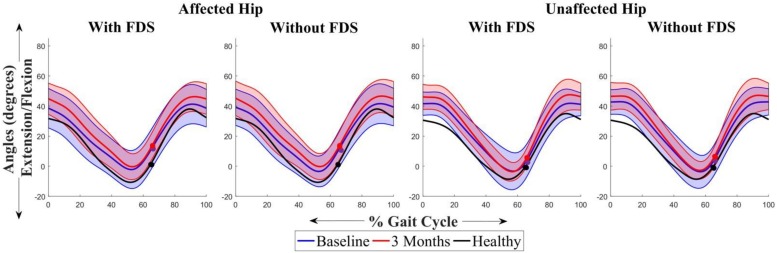
Mean ± Standard deviation (SD) hip plantarflexion/dorsiflexion of the **(A)** affected hip with FDS, **(B)** affected hip without FDS **(C)** unaffected hip with FDS and **(D)** unaffected hip without FDS, at baseline and 3 months of all participants. The black line represents the mean hip plantarflexion/dorsiflexion of one representative healthy child. The dots represent the start of swing phase.

Figure 4 shows the average sagittal plane kinematics of the hip for the unaffected and affected legs with FDS for both sessions. No difference was observed between baseline and 3 months or between with and without FDS at baseline or at 3 months on the affected (Figures 4A,B) and unaffected legs (Figures 4C,D).

### Toe Displacement

**FIGURE 5:**
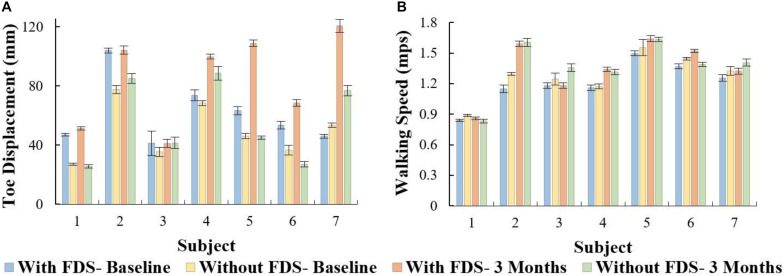
**(A)** Mean ± Standard error of toe displacement of the affected side with and without FDS. **(B)** Mean ± Standard error of walking speed for the affected side with and without FDS.

On comparing toe displacement of the affected leg while walking with and without FDS both at baseline and at 3 months, following results were obtained:

There was no significant effect of duration of use (i.e., from baseline to 3 months [*F*(1,6) = 5.08, *p* > 0.05, *N* = 7], Cohen’s *d* effect size = 0.9) but there was a significant effect of FDS (i.e., with vs. without device [[*F*(1,6) = 18.79, *p* < 0.005, *N* = 7], Cohen’s *d* effect size = 0.76) and there was no significant interaction effect ([*F*(1,6) = 3.58, *p* > 0.05, *N* = 7], Cohen’s *d* effect size = 0.37) using repeated measures ANOVA. At 3 months compared to baseline with FDS, participants showed no significant difference in toe displacement in the affected leg, but participants 1, 4, 5, 6, and 7 increased their mean toe displacement (Figure 5A). Also, at 3 months compared to baseline without FDS (therapeutic effect), participants showed no significant difference [*t*(−6) = 1.44, *p* > 0.05, Cohen’s *d* effect size = 0.54] in the affected leg, but participants 2, 3, 4, and 7 increased their mean toe displacement while subject 6 decreased their mean toe displacement (Figure 5A). At baseline, participants 1, 2, 4, 5, and 6 increased their mean toe displacement in the affected leg when walking with FDS compared to without FDS (orthotic effect), though no significant difference was observed between the two groups after Bonferroni correction [*t*(6) = 2.79, *p* > 0.025, *N* = 7, Cohen’s *d* = 1.05]. At 3 months, there was a significant difference due to the FDS compared to without FDS (orthotic effect) after Bonferroni correction [*t*(6) = 3.54, *p* < 0.025, *N* = 7, Cohen’s *d* = 1.0] on the affected leg, where participants 1, 2, 4, 5, 6, and 7 showed increased toe displacement with FDS.

### Walking Speed

There was no significant main effect of device (i.e., with vs. without device [F(1,6) = 3.71, *p* > 0.05, N = 7], Cohen’s d effect size = 0.78) or duration of use (i.e., baseline to 3 months[(F(1,6) = 5.74, *p* > 0.05, N = 7]), Cohen’s d effect size = 0.9) or interaction effect ([F(1,6) = 3.58, *p* > 0.05, N = 7], Cohen’s d effect size = 0.58) using repeated measures ANOVA. The results show that at 3 months compared to baseline with FDS, participants 2, 4, 5, 6, and 7 increased their mean speed (Figure 5B). Additionally, at 3 months compared to baseline without FDS, participants 2, 3, 4, 5, and 7 increased their speed, and participants 1 and 6 decreased their mean speed (Figure 5B). At baseline, participants 1, 2, 3, 5, 6, and 7 (Figure 5B) showed a decrease in speed with FDS compared to without FDS. At 3 months, Participants 1, 4, and 6 (Figure 5B) increased their mean speed with FDS compared to without FDS, but participants 3 and 7 decreased their speed with FDS compared to without FDS.

### Step Length

There was no significant effect of device or duration of use with the repeated measures ANOVA. The results show that at 3 months compared to baseline with use of FDS, participants 2 and 5 increased their mean step length by over 20 mm (Table 3). Also, at 3 months compared to baseline without the use of FDS, participants 2, 3, 4, and 5 increased their step length by over 20 mm, but participants 1 and 6 decreased their mean step length by over 20 mm (Table 3). At baseline, subject 2 showed an over 20 mm increase in their mean step length (Table 3) when walking without FDS compared to with FDS. At 3 months, participants 3 and 7 showed an over 20 mm increase in their mean step length (Table 3), but subject 6 showed a decrease in mean step length when walking without FDS compared to with FDS.

**TABLE 3:** The table shows the Mean ± Standard error of total time, step length, and swing time of the affected side.

**Subject**	**With FDS- baseline**	**Without FDS- baseline**	**With FDS- 3 Months**	**Without FDS- 3 Months**
Step Length (mm)				
1	488.12±6.14	502.38±5.46	479.92±7.65	470.58±7.33
2	600.14±9.02	623.33±7.19	686.43±14.62	699.90±16.10
3	411.21±11.06	424.52±14.76	427.61±12.24	483.91±18.89
4	553.90±22.10	552.55±7.23	563.95±10.88	572.33±9.16
5	758.83±10.56	762.15±30.92	786.26±15.23	802.34±7.27
6	616.17±6.92	617.94±6.69	621.08±8.06	549.16±9.74
7	676.62±7.15	680.69±10.14	627.80±16.66	675.48±0.019
Total time (seconds)				
1	1.15±0.009	1.12±0.009	1.14±0.013	1.12±0.010
2	1.10±0.022	1.03±0.009	0.94±0.009	0.93±0.020
3	0.89±0.011	0.88±0.024	0.89±0.016	0.85±0.019
4	1.02±0.012	1.01±0.017	0.91±0.014	0.93±0.006
5	1.11±0.008	1.08±0.01	1.07±0.009	1.06±0.01
6	0.94±0.013	0.90±0.007	0.85±0.007	0.87±0.009
7	1.19±0.019	1.13±0.03	1.09±0.017	1.07±0.011
Swing Time (s)				
1	0.44±0.005	0.40±0.004	0.39±0.006	0.38±0.006
2	0.40±0.006	0.38±0.004	0.36±0.003	0.35±0.007
3	0.35±0.006	0.35±0.007	0.33±0.007	0.31±0.01
4	0.33±0.007	0.34±0.008	0.35±0.008	0.34±0.008
5	0.48±0.005	0.45±0.015	0.45±0.004	0.44±0.004
6	0.39±0.005	0.37±0.003	0.34±0.003	0.36±0.005
7	0.38±0.007	0.42±0.013	0.36±0.01	0.36±0.004

### Total Time

There was a significant effect of device and duration of use but there was no interaction effect using the repeated measures ANOVA. The results show that the mean total time at 3 months compared to baseline with the use of FDS (*p* > 0.025, N = 7) did not show a significant effect after Bonferroni correction, but participants 2, 4, and 7 showed a decrease in mean total time (Table 3) of over 0.05 s. The mean total time at 3 months compared to baseline without the use of FDS (*p* < 0.025, N = 7) showed a significant effect after Bonferroni correction, where participants 2, 4, and 7 showed a decrease in mean total time (Table 3) of over 0.05 s. At baseline, there was no significant effect after Bonferroni correction (*p* > 0.025, N = 7) in mean total time between with and without FDS, but participants 1, 2, 5, 6, and 7 showed a decrease in mean total time of over 0.05 s while walking without the FDS compared with FDS (Table 3). At 3 months, there was no significant effect (*p* < 0.025, N = 7) in mean total time while walking without the FDS compared with FDS (Table 3). No participants showed a change in mean total time greater than 0.05 s.

### Swing Time

There was no significant effect of device but there was a significant effect of duration of use but there was no significant interaction effect using the repeated measures ANOVA. No significant change in swing time was observed between baseline and 3 months with the use of FDS (Table 3). The results show that at 3 months compared to baseline with the use of FDS, participants 1, 2, 3, 5, 6, and 7 showed a decrease in mean swing time (Table 3). Also, at 3 months compared to baseline without the use of FDS, participants 1, 2, 3, 5, 6, and 7 showed a decrease in mean swing time (Table 3).

At baseline, the mean swing time increased for participants 1, 2, 5, 6 and decreased for subject 7 with use of FDS compared to without use of FDS (Table 3). At 3 months, the mean swing time increased for participant 3 with use of FDS compared to without use of FDS (Table 3).

### Correlation Between Toe Displacement and Speed

**FIGURE 6:**
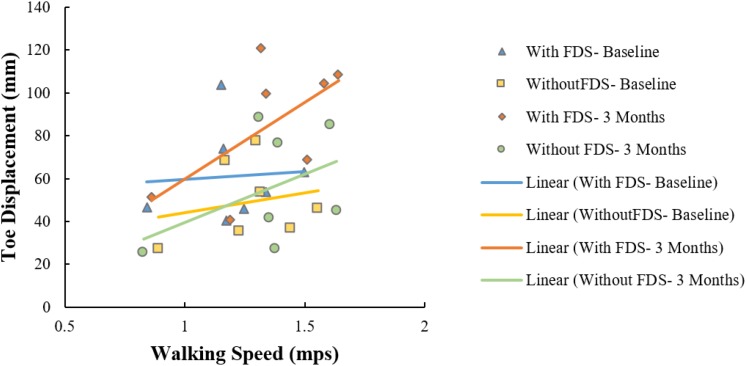
Correlation between toe displacement and speed of the affected side with and without FDS at baseline and 3 months.

All conditions showed no significant Pearson’s *r* correlation between toe displacement and speed (With FDS- Baseline: *r* = 0.07, *p* > 0.05, df = 5 Without FDS- Baseline: *r* = 0.20, *p* > 0.05, df = 5 With FDS- 3 Months: *r* = 0.62, *p* > 0.05, df = 5 Without FDS- 3 Months: *r* = 0.44, *p* > 0.05, df = 5). However, high positive correlation was observed between toe displacement and speed at 3 months with FDS compared to without FDS (Figure 6).

### Temporal and Spatial Symmetry

**FIGURE 7:**
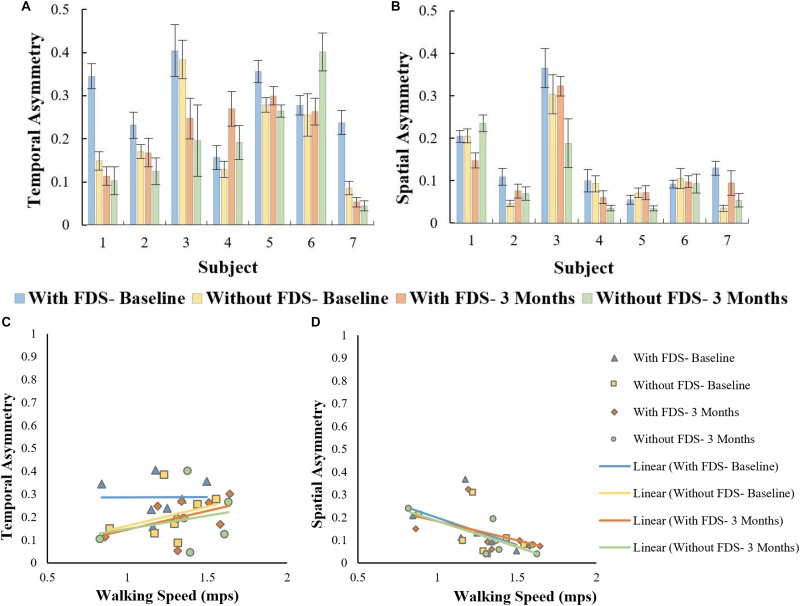
**(A)** Overall asymmetry of the affected side with and without FDS at baseline and 3 months. **(B)** Spatial asymmetry of the affected side with and without FDS at baseline and 3 months. **(C)** Correlation between temporal asymmetry and speed of the affected side with and without FDS at baseline and 3 months. **(D)** Correlation between spatial asymmetry and speed of the affected side with and without FDS at baseline and 3 months.

There was no significant effect of device (i.e., with and without device [*F*(1,6) = 5.724, *p* > 0.005, *N* = 7], Cohen’s *d* effect size = 0.48) or duration of use (i.e., baseline to 3 months[(*F*(1,6) = 1.871, *p* > 0.05, *N* = 7]), Cohen’s *d* effect size = 0.24)and there was no significant ([*F*(1,6) = 3.426, *p* > 0.05, *N* = 7], Cohen’s *d* effect size = 0.4) interaction effect using the repeated measures ANOVA on temporal symmetry (Figure 7a). Overall, temporal asymmetry was higher with FDS at baseline compared to without FDS in participants 1, 2, 5, and 7. At 3 months, subject 4 showed higher asymmetry with FDS compared to without FDS, while subject 6 showed higher asymmetry without FDS compared to with FDS. Participants 2, 3 and 7 showed decreased asymmetry from baseline to 3 months without FDS. Participants 4 and 6 showed increased asymmetry from baseline to 3 months without FDS.

There was no significant effect of device or duration of use using the Friedman Non-parametric test (*p* > 0.05) on spatial symmetry (Figure 7b). Spatial asymmetry was higher with FDS at baseline compared to without FDS for participants 2 and 7. Subject 1 showed decreased asymmetry with FDS at 3 months compared to without FDS, while participants 3, 4, and 5 showed increased asymmetry at 3 months with FDS compared to without FDS. Participants

3, 4, and 5 showed decreased asymmetry from baseline to 3 months without FDS.

No significant Pearson’s *r* correlation was observed between temporal asymmetry and speed in all conditions (With FDS- Baseline [*r* = 0.01, *p* > 0.05, df = 5], with FDS- 3 Months [*r* = 0.49, *p* > 0.05, df = 5] without FDS- Baseline. [*r* = 0.481, *p* > 0.05, df = 5] and without FDS- 3 Months[*r* = 0.261, *p* > 0.05, df = 5]) (Figure 7c).

Significant Pearson’s *r* correlation was observed between spatial asymmetry and speed at without FDS- 3 months [*r* = 0.76, *p* < 0.05, df = 5] (Figure 7d). No significant correlation was observed between spatial asymmetry and speed in the other conditions (With FDS- Baseline [*r* = 0.46, *p* > 0.05], Without FDS- Baseline [*r* = 0.481, *p* > 0.05, df = 5] With FDS- 3 Months [*r* = 0.49, *p* > 0.05, df = 5]).

## Discussion

Children and adolescents with foot drop have difficulty clearing their foot off the floor during swing, hence producing inefficient gait with altered gait kinematics and interlimb symmetry. This, in turn, results in reduced speed and step length, and increased gait cycle duration when walking. Current research is focused on reducing these deficits with the use of FDS, and on understanding both the orthotic and therapeutic effects of FDS. In this study, the efficacy of FDS usage for 3-month was investigated by evaluating the changes in mechanistic outcomes such as kinematics, inter-limb asymmetry, and functional outcomes such as speed, step length, and gait cycle duration. This study evaluates mechanistic parameters, using outcomes such as symmetry and toe displacement, which have not been considered in other research investigating FDS in children. Following the use of FDS, increased dorsiflexion of the ankle was observed, aiding in foot clearance. While no significant increase in speed or step length was found, a correlation between toe displacement and speed, and between spatial asymmetry and speed was observed.

The results showed that participants increased their toe displacement with FDS compared to without FDS, demonstrating that the FDS device is assisting in clearing the foot, as shown in Figure 5A. This is further supported by the mean dorsiflexion angle during swing (Figure 2), where the mean ankle angle with FDS is greater than the angle without FDS by over 3 degrees, as Winter et al. showed that a small change in dorsiflexion angle could have a considerable effect on toe displacement (Winter, 1992). These results are also in accordance with the results observed by other researchers (Van Der Linden et al., 2008; Prosser et al., 2012; Damiano et al., 2013), who found that using FDS produced increased dorsiflexion during swing phase in children. Further, a similar orthotic effect of increased dorsiflexion was also observed with percutaneous electrical stimulation (Pierce et al., 2004a,b; Postans and Granat, 2005). Additionally, an adaptive effect was observed from baseline to 3 months, where both mean dorsiflexion and toe displacement increased with the use of FDS. This could be due to the participants adapting to the FDS with time. This effect may partially explain the decrease in temporal and spatial symmetry, and walking speed with FDS compared to without FDS in some of the participants at baseline.

Though some of the participants showed an increase in walking speed at the 3-month visit when walking with the FDS compared to without FDS, they did not exhibit any statistically significant change in walking speed between conditions as shown in Figure 5B. This result is in concordance with Prosser et al., where they showed an increase in dorsiflexion angle but no statistically significant change in walking speed after a 3-month intervention with FES in “free- and fast-speed” trials (Prosser et al., 2012). Similar results were also observed by Orlin et al. with percutaneous stimulation, where they achieved improvements in kinematics but no significant change in walking speed (Orlin et al., 2005). Bertoti et al. reported a step length increase in a child with CP after 7 months of gait training with percutaneous stimulation, but no change in walking speed (Bertoti et al., 1997). One possible explanation for the lack of significant changes in walking speed could be that most of the participants in the study were already ambulating with a healthy walking speed at the time of enrollment, which limited further significant improvement. Unlike results from research in adults with hemiplegia post-stroke, where adult participants presented with slower walking speeds and have shown significant increases in speed after the use of FDS (Burridge et al., 1997). Based on our study, we can infer that walking speed in itself might not be an indicator of the deficit or of the recovery in certain populations with walking impairments. Hence, other mechanistic outcomes need to be used to better understand the deficit and recovery that may take place.

A positive correlation (*r* = 0.62) between toe displacement and speed was observed with the use of FDS and a moderate correlation (*r* = 0.44) without FDS at 3 months. This could indicate that an increase in toe displacement may be a contributing factor to the observed increase in walking speed. These results were not statistically significant which could be due to the small sample size, however, the trend could be clinically important. After continuous use of the FDS, some participants increased mean ankle dorsiflexion even while walking without the FDS from baseline to 3 months during the swing phase (Figure 2B), and 4 out of 7 participants increased toe displacement (Figure 5). These effects, however, were not statistically significant and also were not observed in all participants. These clinical improvements while walking without FDS in some participants may indicate that continuous use of the FDS may have resulted in a therapeutic effect but a larger sample is required to further conclusively prove the effect of duration of use.

Spatial asymmetry and speed showed a negative correlation in all conditions, suggesting that an increase in speed is associated with a decrease in spatial asymmetry. The negative correlation was higher at 3 months compared to baseline indicating that a decrease in asymmetry could have contributed to the increase in speed. Though there was no change in step length on the affected side, the symmetry between the two legs improved, indicating that the step length of both legs became more equivalent. Studies on hemiparetic gait have reported that temporal asymmetry is more of a predictor of walking speed than spatial asymmetry (Lin et al., 2006; Balasubramanian et al., 2007; Patterson et al., 2008, 2010; Nadeau, 2014); however, in this study, temporal asymmetry showed a lower correlation with speed compared to spatial asymmetry.

Prior to the start of the study, all the participants were exclusively relying on an AFO for community ambulation to prevent foot drop. In growing children and adolescents, AFOs needs to be fabricated multiple times, thus increasing the incurred expense over time. On the contrary, a FDS device that can be affordably adjusted to accommodate for user growth rather than being periodically replaced might be a more cost-effective solution for foot drop. In addition, the results of this study indicate the potential of FDS to providing not only an orthotic effect to the wearer but also a therapeutic effect as well, without the ROM restriction of an AFO.

A potential limitation of the current study is that participant activity in the community was not monitored. It is possible that increased activity in the community while wearing the FDS device could provide additional therapeutic advantage over time since increased steps would positively correlate with increased stimulation dosing. Researchers did not want to adjust participant behavior so no instructions were provided to increase community ambulation, instructions for community utilization were to wear the FDS device as much as possible while at home and in the community. Additionally, FDS device compliance and usage was not standardized throughout the investigation. Therefore, the individual clinical benefits from wearing the device may be related to device adoption or use. The combination between community ambulation and device usage is important to consider when evaluating the clinical benefits of FDS. In this pilot investigation, evaluating the effect of FDS in pediatric population, we were able to begin to identify relevant outcomes to consider in pediatrics when deciding if a FDS is appropriate for clinical use. Another limitation of this research is the limited sample size. The data indicated some promising results for orthotic and therapeutic effects that should continue to be explored in a larger sample.

## Conclusion

This study indicates that there is an orthotic effect of increased dorsiflexion and toe displacement during swing with the use of the FDS in children with hemiplegia. Further, the study suggests that there could be a potential long-term effect of increased dorsiflexion during swing with continuous use of FDS. The current results are promising, but future studies with a larger sample in a controlled environment would be required to further understand the efficacy of the FDS in children and adolescents and to confirm any training effect conclusively.

## Ethics Statement

This study was carried out in accordance with the recommendations of Kessler Foundation IRB with written informed consent from all subjects. All subjects gave written informed consent in accordance with the Declaration of Helsinki. The protocol was approved by the Kessler Foundation IRB.

## Author Contributions

KN designed the study with assistance from KB and JC. KK, NE, and KN collected the data. KK, RP, and NE analyzed the data. KK drafted the manuscript. RP, NE, KB, JC, and KN revised, reviewed, and finalized the manuscript.

## Conflict of Interest Statement

The authors declare that the research was conducted in the absence of any commercial or financial relationships that could be construed as a potential conflict of interest.
